# The maintenance of polygenic sex determination depends on the dominance of fitness effects which are predictive of the role of sexual antagonism

**DOI:** 10.1093/g3journal/jkab149

**Published:** 2021-04-30

**Authors:** Richard P Meisel

**Affiliations:** Department of Biology and Biochemistry, University of Houston, Houston, TX 77204-5001, USA

**Keywords:** sex chromosomes, house fly, sexual conflict, recessive, additive, overdominant, Genetics of Sex

## Abstract

In species with polygenic sex determination (PSD), multiple male- and female-determining loci on different proto-sex chromosomes segregate as polymorphisms within populations. The extent to which these polymorphisms are at stable equilibria is not yet resolved. Previous work demonstrated that PSD is most likely to be maintained as a stable polymorphism when the proto-sex chromosomes have opposite (sexually antagonistic) fitness effects in males and females. However, these models usually consider PSD systems with only two proto-sex chromosomes, or they do not broadly consider the dominance of the alleles under selection. To address these shortcomings, I used forward population genetic simulations to identify selection pressures that can maintain PSD under different dominance scenarios in a system with more than two proto-sex chromosomes (modeled after the house fly). I found that overdominant fitness effects of male-determining proto-Y chromosomes are more likely to maintain PSD than dominant, recessive, or additive fitness effects. The overdominant fitness effects that maintain PSD tend to have proto-Y chromosomes with sexually antagonistic effects (male-beneficial and female-detrimental). In contrast, dominant fitness effects that maintain PSD tend to have sexually antagonistic multi-chromosomal genotypes, but the individual proto-sex chromosomes do not have sexually antagonistic effects. These results demonstrate that sexual antagonism can be an emergent property of the multi-chromosome genotype without individual sexually antagonistic chromosomes. My results further illustrate how the dominance of fitness effects has consequences for both the likelihood that PSD will be maintained as well as the role sexually antagonistic selection is expected to play in maintaining the polymorphism.

## Introduction

Sex determination is the developmental process by which a genetic signal or environmental cue initiates sexually dimorphic gene regulatory pathways to produce phenotypically different males and females (Bachtrog *et al.* 2014). The master regulators of sex determination evolve fast, often differing between closely related species (Bull 1983; Wilkins 1995; Beukeboom and Perrin 2014). Master regulators can be found on sex chromosomes, and the evolutionary transitions of sex determiners can drive turnover of the sex chromosomes (Abbott *et al.* 2017). These evolutionary transitions include a period of polygenic sex determination (PSD), during which multiple master sex-determining loci on different chromosomes segregate as polymorphisms within species (Moore and Roberts 2013). Understanding the population dynamics of PSD is informative of the factors responsible for the evolutionary divergence of sex determination pathways and sex chromosomes.

PSD has been observed in multiple animal and plant species (Moore and Roberts 2013; Bachtrog *et al.* 2014; Beukeboom and Perrin 2014). Most population genetic models predict that PSD will be an unstable intermediate between monogenic equilibria (Rice 1986; van Doorn and Kirkpatrick 2007). Models that do allow for stable PSD often predict that opposing selection pressures in males and females (*i.e.*, sexually antagonistic selection) may be important for maintaining the polymorphism (Bull 1983; Rice 1986; van Doorn and Kirkpatrick 2007; Meisel *et al.* 2016). It is also possible that variable selection pressures across heterogeneous environments could maintain PSD (Bateman and Anholt 2017).

Most previous theoretical treatments of the selection pressures that maintain PSD considered specific cases with a small number of sex chromosomes segregating within a population. For example, in the platyfish, *Xiphophorus maculatus*, a single chromosome can be an X, Y, or Z (Kallman 1973). Orzack et al. (1980) showed that this polymorphism is maintained because heterogametic females (WY or WX) and males (XY) have higher fitness than their homogametic counterparts (XX females or YY males). In addition, van Doorn and Kirkpatrick (2007, 2010) found that sexually antagonistic selection could maintain a polymorphism in which one chromosome segregates as an XY pair and another chromosome is either an XY or ZW pair. However, these models do not capture the full diversity of PSD, which can include more than two XY or ZW pairs segregating within a single species (Moore and Roberts 2013).

Previous models also did not broadly consider how the maintenance of PSD depends on the dominance of fitness effects. The dominance of sexually antagonistic alleles affects their ability to segregate as stable polymorphisms (Kidwell *et al.* 1977; Connallon and Chenoweth 2019), suggesting that dominance may be important for the maintenance of PSD under sexually antagonistic selection. Dominance also differentially affects the evolutionary fate of X-linked and autosomal alleles that experience sex-specific selection pressures (Rice 1984; Charlesworth *et al.* 1987; Fry 2010; Connallon *et al.* 2012). In a species with PSD, there are proto-sex chromosomes that behave neither like autosomes nor like conventional heteromorphic sex chromosomes; proto-sex chromosomes are diploid in both sexes (like autosomes) but have sex-biased modes of inheritance (like conventional sex chromosomes). This is superficially similar to freely recombining pseudoautosomal regions (PARs) on sex chromosomes (Otto *et al.* 2011), except that in PSD a proto-Y (or proto-W) chromosome can be directly carried by females (males) without X-Y (or Z-W) recombination. Therefore, the expected effects of dominance and sexual antagonism on the evolution of PSD cannot be determined from existing models of autosomal or sex-linked alleles.

The house fly, *Musca domestica*, is a well-suited organism for investigating the maintenance of complex PSD because it has a highly polymorphic sex determination system (Hamm *et al.* 2015). The *M. domestica male determiner* (*Mdmd*) can be found on at least four of the six house fly chromosomes in natural populations (Sharma *et al.* 2017). *Mdmd* causes the house fly ortholog of *transformer* (*Md-tra*) to be spliced into a nonfunctional isoform, which leads to male-specific splicing and expression of downstream genes in the sex determination pathway, thereby producing fertile males (Hediger *et al.* 2010). In the absence of *Mdmd*, *Md-tra* is spliced into a female-determining transcript that regulates the splicing of downstream targets, which promote the development of female morphological and behavioral traits (Hediger *et al.* 2004; Meier *et al.* 2013).

There are two common male-determining proto-Y chromosomes and one female-determining proto-W chromosome in house fly populations. *Mdmd* is most commonly found on the Y (Y^M^) and third (III^M^) chromosomes in natural populations (Hamm *et al.* 2015). Both Y^M^ and III^M^ are young proto-Y chromosomes that arose recently and are nearly identical in gene content to their homologous proto-X chromosomes, known as X and III (Meisel *et al.* 2017; Son and Meisel 2021). In addition, there is a dominant allele of *Md-tra* that is found in many house fly populations (*Md-tra^D^*), which is resistant to *Mdmd* and causes female development regardless of whether there are copies of *Mdmd* in the genotype (McDonald *et al.* 1978; Hediger *et al.* 2010). *Md-tra* is found on the fourth chromosome, and thus fourth chromosomes carrying *Md-tra^D^* (which I will refer to as IV^F^) are W chromosomes. All sequences of *Md-tra^D^* from around the world are identical (Scott *et al.* 2014), suggesting that *Md-tra^D^* arose recently and IV^F^ is a young proto-W chromosome. In populations where Y^M^, III^M^, and *Md-tra^D^* all segregate, there are 18 possible sex chromosome genotypes (Table 1).

**Table 1:** House fly sex chromosome genotypes

	Genotype			
	X or Y^M^	III or III^M^	IV or IV^F^	Sex
*f*_1_	X/X	III/III	IV/IV	Female
*f*_2_	X/X	III/III	IV/IV^F^	Female
*f*_3_	X/X	III/III^M^	IV/IV^F^	Female
*f*_4_	X/X	III^M^/III^M^	IV/IV^F^	Female
*f*_5_	X/Y^M^	III/III	IV/IV^F^	Female
*f*_6_	X/Y^M^	III/III^M^	IV/IV^F^	Female
*f*_7_	X/Y^M^	III^M^/III^M^	IV/IV^F^	Female
*f*_8_	Y^M^/Y^M^	III/III	IV/IV^F^	Female
*f*_9_	Y^M^/Y^M^	III/III^M^	IV/IV^F^	Female
*f*_10_	Y^M^/Y^M^	III^M^/III^M^	IV/IV^F^	Female
*m*_1_	X/X	III/III^M^	IV/IV	Male
*m*_2_	X/X	III^M^/III^M^	IV/IV	Male
*m*_3_	X/Y^M^	III/III	IV/IV	Male
*m*_4_	X/Y^M^	III/III^M^	IV/IV	Male
*m*_5_	X/Y^M^	III^M^/III^M^	IV/IV	Male
*m*_6_	Y^M^/Y^M^	III/III	IV/IV	Male
*m*_7_	Y^M^/Y^M^	III/III^M^	IV/IV	Male
*m*_8_	Y^M^/Y^M^	III^M^/III^M^	IV/IV	Male

There is evidence that natural selection maintains PSD in house fly populations. First, the proto-Y chromosomes have remained at stable frequencies within house fly populations over decades (Kozielska *et al.* 2008; Meisel *et al.* 2016). Second, Y^M^ and III^M^ are distributed along north–south clines on multiple continents (Denholm *et al.* 1986; Tomita and Wada 1989; Hamm *et al.* 2005; Kozielska *et al.* 2008), and temperature is the best predictor of their frequencies (Feldmeyer *et al.* 2008). This suggests that heterogenous (temperature-dependent) selection pressures maintain the Y^M^ and III^M^ chromosomes across populations. The stable frequencies within populations, yet divergent frequencies across populations, suggest that there are specific fitness effects associated with the house fly proto-Y chromosomes within each population. Third, males carrying multiple proto-Y chromosomes (*e.g.*, both Y^M^ and III^M^, or two copies of III^M^) can be found in some populations (*e.g.*, Hamm and Scott 2009). The frequency of *Md-tra^D^* is positively correlated with the frequency of these multi-Y males across populations, suggesting that selection for balanced sex ratios may further maintain PSD (Meisel *et al.* 2016).

The rich body of observational data in house fly makes it a well-suited system around which to develop population genetic models to determine how PSD can be maintained in natural populations. For example, Kozielska et al. (2006) showed that, even in the complex house fly PSD system, sex ratios are not expected to deviate substantially from 1:1 male:female. In addition, despite no experimental evidence for sexually antagonistic effects of any house fly proto-sex chromosomes, another population genetic model demonstrated that the frequencies of the proto-sex chromosomes in natural populations are consistent with sexually antagonistic selection maintaining the polymorphism (Meisel *et al.* 2016). However, that model assumed that the proto-Y chromosomes (Y^M^ and III^M^) have additive fitness effects, which may not be true if, for example, beneficial mutations are recessive or dominant (Orr 2010). In addition, Y chromosomes are expected to carry recessive deleterious alleles (Charlesworth and Charlesworth 2000), which can further violate the assumption of additive fitness effects. To address the shortcomings of previous models, I used population genetic simulations to investigate whether sex chromosomes with nonadditive fitness effects can maintain the complex house fly PSD system. I specifically tested if these selection pressures can produce proto-sex chromosome frequencies similar to those observed in natural populations of house fly, and I determined the conditions under which sexually antagonistic effects of those proto-sex chromosomes are expected.

## Materials and methods

**Figure 1:**
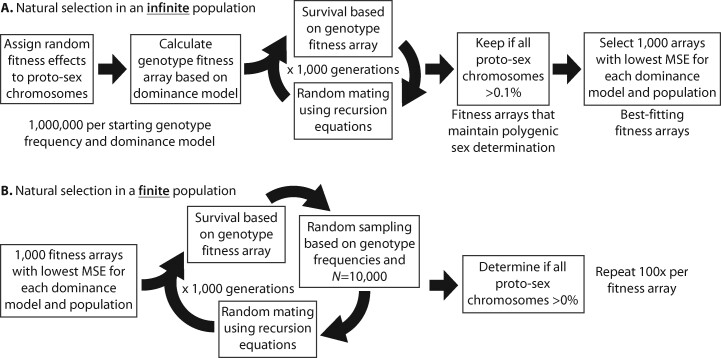
Steps in forward population genetic simulations with (A) infinite or (B) finite populations. The best-fitting arrays are those with the lowest mean MSE comparing simulated proto-sex chromosome frequencies to those observed in each natural population.

I used a simulation approach (Figure 1A) to identify fitness effects of two proto-Y chromosomes (Y^M^ and III^M^) and one proto-W chromosome (IV^F^) that maintain PSD under four different dominance scenarios. I used simulations to test if fitness effects maintain PSD because the model parameters were too complex to analytically solve for equilibrium conditions. To do so, I first assigned sex-specific fitness effects, $s_{ij}$, for each j proto-Y or proto-W chromosome in each sex i by drawing from a random uniform distribution between −1 and 1 (negative values indicate beneficial effects, and positive indicate deleterious). I then used those fitness effects to calculate single chromosome genotype fitness values assuming either additive, dominant, recessive, or overdominant (*i.e.*, heterozygote advantage) fitness effects of the proto-Y chromosomes (Table 2). I calculated the fitness of each of the 18 multi-chromosome genotypes (Table 1) by multiplying all single chromosome fitness values.

**Table 2:** Single proto-Y/proto-X chromosome genotype fitness values with different dominance scenarios

	XX	XY	YY
Additive			
$\;s_{ij}>0$	$1-s_{ij}$	$1-0.5s_{ij}$	1
$\;s_{ij}<0$	1	$1+0.5s_{ij}$	$1+s_{ij}$
Dominant			
$\;s_{ij}>0$	$1-s_{ij}$	1	1
$\;s_{ij}<0$	1	$1+s_{ij}$	$1+s_{ij}$
Recessive			
$\;s_{ij}>0$	$1-s_{ij}$	$1-s_{ij}$	1
$\;s_{ij}<0$	1	1	$1+s_{ij}$
Overdominant (male only)			
$\;s_{ij}>0$	$1-s_{ij}$	1	$1-s_{ij}$
$\;s_{ij}<0$	$1+s_{ij}$	1	$1+s_{ij}$

Calculations are shown for determining single chromosome genotype fitness values from the selective effect $s_{ij}$ of proto-Y chromosome j in sex i. Three genotypes are shown (XX, XY, and YY). The Y chromosome could either be Y^M^ or III^M^, and the X chromosome could either be X or III.

To test if the fitness effects result in stable PSD, I determined the frequency of each genotype and proto-sex chromosome after 1000 generations of random mating with the assigned fitness values (Figure 1A). Simulations were started with either equal frequencies of all 18 genotypes or the genotype frequencies observed in one of three natural populations (CA, NC, or NY) sampled from North America (Hamm and Scott 2008; Scott *et al.* 2013; Meisel *et al.* 2016). I performed simulations for one million different fitness values for each set of starting genotype frequencies and each dominance model using previously developed recursion equations (Hamm 2008; Meisel *et al.* 2016). I retained fitness effects in which all three proto-sex chromosomes remained polymorphic at a frequency >0.1% after 1000 generations. From those, I selected the 1000 fitness effects with the smallest mean squared error (MSE) between simulated proto-sex frequencies and those observed in each natural population (CA, NC, or NY) for each dominance model, population, and starting genotype frequency (Figure 1A). In doing so, I am essentially applying an approximate Bayesian computation approach to fit selection pressures to natural populations (Beaumont *et al.* 2002). I observed the same general trends regardless of the starting genotype frequencies, and I only present the results of simulations starting with equal frequencies in the main text (other results are presented in the Supplementary Material). I additionally evaluated how well those fitness effects maintain PSD in a finite population of 10,000 individuals (Figure 1B). I also selected 1000 random fitness effects for each set of starting genotype frequencies and each dominance model to determine null expectations under my model. Additional details are provided in the Supplementary Methods.

### Data availability

Supplementary material is available at figshare: https://doi.org/10.25387/g3.14488854. Results underlying this article are available in the article and in its online Supplementary material.

## Results

### Dominance of fitness effects and the maintenance of PSD

**Figure 2:**
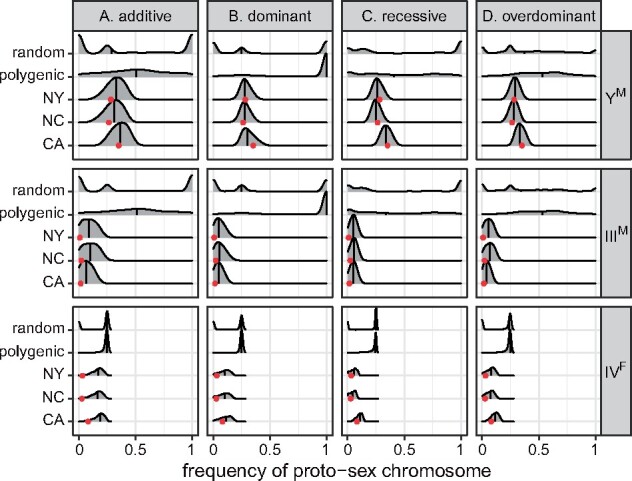
Smoothed histograms show the frequency of each proto-sex chromosome (Y^M^, III^M^, and IV^F^) after 1000 generations in simulations using either 1000 random fitness arrays (random), fitness arrays that maintain PSD (polygenic), or the 1000 best-fitting fitness arrays for each population (CA, NC, or NY). Simulations were started with equal frequencies of all genotypes. The vertical line within each histogram shows the median. Red dots show the observed proto-sex chromosome frequencies in each natural population. Fitness arrays were calculated assuming either (A) additive, (B) dominant, (C) recessive, or (D) overdominant fitness effects of the proto-Y chromosomes.

I find that the probability that PSD is maintained (with a frequency of each proto-sex chromosome >0.1% after 1000 generations) is affected by the dominance of the fitness effects of the Y^M^ and III^M^ proto-Y chromosomes (Supplementary Figures S1A and S2A). When fitness effects of proto-Y chromosomes are additive, <5% of fitness values maintain PSD. In contrast, when fitness effects of the proto-Y chromosomes are recessive, ∼20% of fitness values maintain PSD. Overdominant fitness effects are the most likely to maintain PSD, with >40% of fitness values maintaining proto-sex chromosomes at a frequency >0.1% after 1000 generations. When additive, recessive, or overdominant (but not dominant) fitness effects maintain PSD, there is a broad range of frequencies at which the proto-Y chromosomes (Y^M^ and III^M^) can segregate as polymorphisms (Figure 2 and Supplementary Figure S3). Randomly chosen fitness effects, in comparison, tend to produce one of three proto-Y chromosome frequency classes: 0 (loss of a proto-Y, because a different XY system has taken over), 1 (fixation of the proto-Y chromosomes, corresponding to a monogenic ZW system), and 0.25 (the expectation for a monogenic XY system).

Most dominant fitness effects that appear to maintain PSD are paths to the eventual fixation of the proto-Y chromosomes. More than 25% of dominant fitness maintain PSD for 1000 generations (Supplementary Figures S1A and S2A), but most of those produce very high frequencies of the Y^M^ and III^M^ chromosomes (Figure 2B and Supplementary Figure S3). A proto-Y chromosome with a dominant fitness effect segregating at very high frequency is consistent with a beneficial effect that masks the recessive variant (in this case the homologous proto-X), allowing the proto-X chromosome to persist at a low frequency for a long time (Hartl and Clark 2007). To test this prediction, I selected fitness effects that maintain PSD for 1000 generations, and I ran simulations with those fitness effects for 1,000,000 generations. I considered a proto-Y chromosome to reach “fixation” after 1,000,000 generations if it achieves a frequency >99.9% (*i.e.*, the same threshold for maintaining PSD after 1000 generations). Consistent with the prediction, >50% of dominant fitness effects that maintain PSD for 1000 generations eventually cause the Y^M^ or III^M^ proto-Y chromosomes to reach fixation within 1,000,000 generations, regardless of the starting genotype frequencies (Supplementary Figures S4–S7). In contrast, ≤1% of additive, recessive, or overdominant fitness effects of proto-Y chromosomes that maintain PSD for 1000 generations eventually result in fixation of a proto-Y chromosome after 1,000,000 generations. Despite the eventual fixation of proto-Y chromosomes with many dominant fitness effects, there are subset of dominant fitness effects that maintain Y^M^ and III^M^ at lower frequencies, resembling those observed in natural populations (Figure 2B). For these dominant fitness effects that produce proto-sex chromosome frequencies similar natural populations, fixation of proto-Y chromosomes never occurs after 1,000,000 generations (Supplementary Figures S4–S7). Therefore, dominant fitness effects can maintain PSD at equilibrium, although most dominant fitness effects result in fixation or loss of the proto-Y chromosomes. Below, I describe how dominant fitness effects that can or cannot maintain PSD differ in their signatures of sexual antagonism.

### Evaluating if selection pressures can produce proto-sex chromosome frequencies observed in natural populations

Selecting only the 1000 fitness effects that produce proto-sex chromosome frequencies most similar to natural populations further affects the relationships between dominance and proto-sex chromosome frequencies. For example, dominant, recessive, and overdominant fitness effects can produce proto-sex chromosome frequencies that are more similar to those in natural populations when compared with additive fitness effects (Figure 2 and Supplementary Figures S1–S3). The poorer fit of additive models arises because they struggle to produce frequencies of III^M^ and IV^F^ as low as those observed in the CA, NC, and NY populations (Figure 2 and Supplementary Figure S3).

The low frequency of the III^M^ chromosome in the CA, NC, and NY populations has consequences for the ability of natural selection to maintain the polymorphism. As above, I define loss of the III^M^ chromosome (or fixation of the III chromosome) as a frequency below 0.1% (or above 99.9% for III). Additive fitness effects that maintain the III^M^ chromosome at frequencies similar to those in CA, NC, or NY for 1000 generations often (9–17% of the time) result in the loss of III^M^ within 1,000,000 generations (Supplementary Figures S8 and S9). In contrast, <7% of dominant, recessive, or overdominant fitness effects that maintain III^M^ at frequencies observed in natural populations for 1000 generations lead to the eventual loss of III^M^ within 1,000,000 generations. III^M^ is especially likely to be lost within 1,000,000 generations when it is segregating at a lower frequency after 1000 generations, regardless of the dominance of fitness effects (Supplementary Figures S8 and S9). The Y^M^ chromosome, in comparison, segregates at a higher frequency that III^M^ all three populations (Figure 2B). Y^M^ is lost in ≤2% of simulations after 1,000,000 generations when I apply fitness effects that maintain proto-sex chromosomes at frequencies similar to those observed in natural populations, regardless of the dominance of fitness effects (Supplementary Figure S10). I therefore conclude that low frequency proto-Y chromosomes, such as III^M^, are difficult to maintain as polymorphisms under the model I have applied here. Moderate frequency proto-Y chromosomes, such as Y^M^, on the other hand, can be maintained under a variety of dominance scenarios.

The majority of fitness effects that maintain PSD cause IV^F^ to segregate at a frequency of 25%, regardless of the dominance of the proto-Y chromosomes (Figure 2 and Supplementary Figure S3). A frequency of 25% is the expectation for a W chromosome in a randomly mating population with monogenic ZW sex determination. Therefore, in a system with two proto-Y chromosomes and one proto-W, PSD is expected to be maintained by the (near) fixation of the ZW female genotype. In contrast, IV^F^ makes up 2–9% of all fourth chromosomes in the CA, NC, and NY populations (Hamm and Scott 2008; Scott *et al.* 2013; Meisel *et al.* 2016). A subset of fitness arrays in my simulations maintain IV^F^ at <25% for all dominance models, including those that produce proto-sex chromosome frequencies most similar to those observed in natural populations (Figure 2 and Supplementary Figure S3). It is thus possible for PSD to be maintained with a single proto-W chromosome at a frequency <25%. However, the frequency of IV^F^ is still greater in simulated populations than in natural populations (Figure 2).

### Selection pressures can maintain PSD in finite populations

I next used simulations to examine how well natural selection maintains PSD when population sizes are finite (*N *=* *10^4^ individuals). I calculated the proportion of simulations (out of 100) in which a given proto-sex chromosome reached fixation in a finite population for each fitness array to estimate of the probability of fixation ($P_{\mathrm{fix}}$). The $P_{\mathrm{fix}}$ value for a given proto-Y or proto-W chromosome (Y^M^, III^M^, or IV^F^) is equal to the probability of loss ($P_{\mathrm{loss}}$) of the homologous proto-X or proto-Z (X, III, or IV), and *vice versa*. I also simulated finite populations of the same size but without selection (*i.e.*, genetic drift only). I used Fisher’s exact test to determine if the $P_{\mathrm{fix}}$ or $P_{\mathrm{loss}}$ of a proto-sex chromosome is significantly different between populations with and without selection.

**Figure 3:**
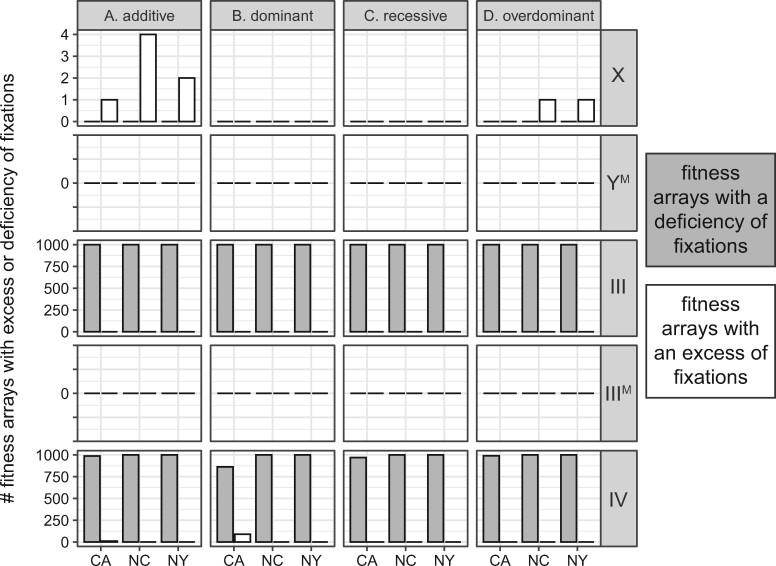
Bar plots show the number of fitness arrays that result in a deficiency (gray) or excess (white) of fixations of each proto-sex chromosome (X, Y^M^, III, III^M^, or IV) in a finite population, relative to simulations in which there are no fitness differences across proto-sex chromosomes (*i.e.*, drift only). The fitness arrays are the 1000 best-fitting fitness values for each population (CA, NC, or NY) when simulations were started with equal frequencies of all genotypes. Fitness effects of the proto-Y chromosomes are either (A) additive, (B) dominant, (C) recessive, or (D) overdominant.

When a proto-sex chromosome is at low frequency, selection pressures that maintain PSD decrease $P_{\mathrm{fix}}$ and $P_{\mathrm{loss}}$ in finite populations, relative to populations without selection. For example, III^M^ is rare in all three populations, and it is the most likely proto-sex chromosome to be lost when population size is finite (Supplementary Figures S11 and S12). In turn, selection pressures that maintain PSD, decrease $P_{\mathrm{fix}}$ of chromosome III relative to drift alone (Figure 3 and Supplementary Figure S13), which is equivalent to decreasing $P_{\mathrm{loss}}$ of III^M^. The IV^F^ proto-W chromosome is also at a low frequency in all three populations, and the maintenance of the IV^F^ polymorphism (*i.e.*, the standard IV chromosome does not fix) is substantially greater when there is selection (Figure 3 and Supplementary Figure S13). Therefore, when loss via drift is likely because a proto-sex chromosome is at low frequency (such as III^M^ and IV^F^), selection pressures that maintain PSD decrease the probability of loss.

A different effect is observed for sex chromosomes segregating at higher frequencies. The X and Y^M^ chromosomes rarely fix in finite populations with selection (Supplementary Figures S11 and S12). Y^M^ is at a higher frequency than III^M^ and IV^F^ across all three populations (Meisel *et al.* 2016), and the high frequency of Y^M^ is likely responsible for its low $P_{\mathrm{loss}}$ within 1000 generations. However, for a small fraction of fitness effects that maintain proto-sex chromosomes at frequencies similar to those in natural populations (<10%), $P_{\mathrm{fix}}$ for both the X and Y^M^ chromosomes is greater with selection and drift than under drift alone (Figure 3). The slightly elevated $P_{\mathrm{fix}}$ of the X and Y^M^ chromosomes with selection is observed for all four dominance models (Supplementary Figure S13). Therefore, when fixation or loss is unlikely via drift (because the proto-Y chromosome is at high frequency), selection pressures that maintain PSD do not decrease $P_{\mathrm{fix}}$ or $P_{\mathrm{loss}}$ of a proto-sex chromosome in a finite population.

### Sexual antagonism and the maintenance of PSD

Sexually antagonistic selection is predicted to maintain PSD (Bull 1983; Rice 1986; van Doorn and Kirkpatrick 2007; Meisel *et al.* 2016). A negative intersexual fitness correlation is a hallmark of sexually antagonistic genetic variation (Bonduriansky and Chenoweth 2009; Chippindale *et al.* 2001; Rice and Chippindale 2001). I therefore inspected if there is a negative correlation between male and female fitness when PSD is maintained. Each of the eight male house fly proto-sex chromosome genotypes has a corresponding female genotype that differs only because females carry a copy of the IV^F^ proto-W chromosome and males do not (Table 1). There are two additional female genotypes (X/X; III/III; IV/IV and X/X; III/III; IV^F^/IV) that do not have a corresponding male genotype because they do not have a proto-Y chromosome. To test for sexually antagonistic multi-chromosome genotypes, I calculated Spearman’s rank order correlation of fitness between males and females ($\rho_{MF}$) for the eight pairs of male and female genotypes in each fitness array.

**Figure 4:**
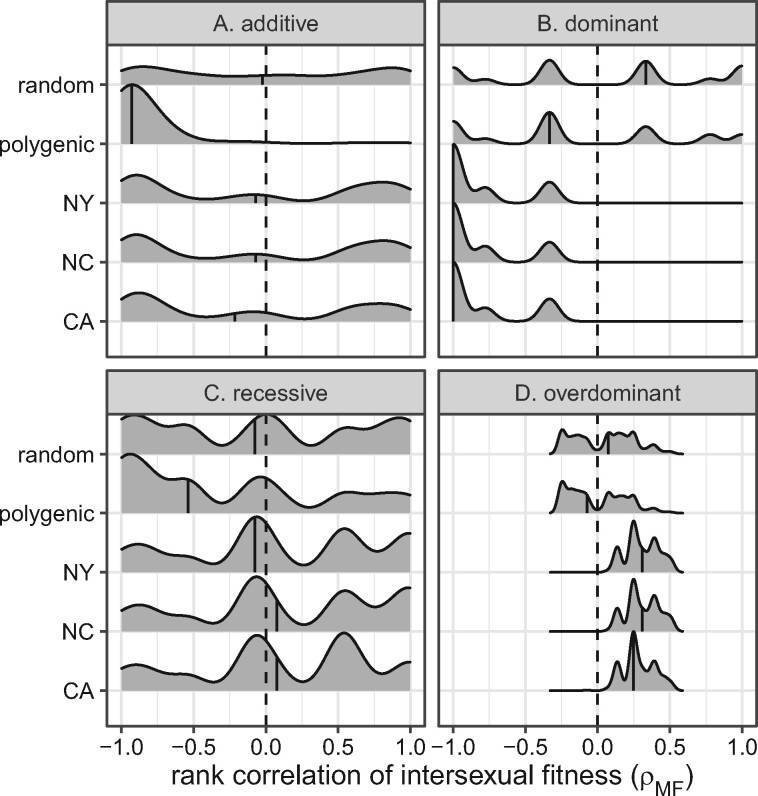
Smoothed histograms show distributions of intersexual fitness correlations ($\rho_{MF}$) for genotype fitness arrays used in simulations started with equal frequencies of all genotypes. Correlations are shown for the 1000 best-fitting genotypic fitness arrays for the CA, NC, and NY populations. Correlations are also shown for all fitness arrays that maintain PSD (polygenic), as well as 1000 random fitness arrays. Dashed vertical lines show a correlation of 0, and solid vertical lines within histograms show the median.

Additive fitness effects that maintain PSD (*i.e*., all proto-sex chromosomes at frequency >0.1%) tend to have $\rho_{MF}<0$ (Figure 4A and Supplementary Figure S14). However, when proto-sex chromosome frequencies resemble those in natural populations and fitness effects are additive, $\rho_{MF}$ can be either positive or negative. There is also no consistent signal of $\rho_{MF}<0$ for random fitness effects, regardless of dominance (Figure 4 and Supplementary Figure S14), demonstrating that $\rho_{MF}<0$ is not an intrinsic property of the model. In comparison, $\rho_{MF}<0$ when I previously applied a model with additive fitness effects both within and across chromosomes (Meisel *et al.* 2016). In the model presented here, multi-chromosome genotype fitness is calculated as the product of single chromosome genotype fitness values, rather than by summing across chromosomes. Therefore, there is some evidence that additive fitness effects that maintain PSD result in $\rho_{MF}<0$, but it depends on specifics of how the model is parameterized. I address the causes and effects of $\rho_{MF}<0$ in more detail in the following section.

Intersexual fitness correlations tend to be negative ($\rho_{MF}<0$) when fitness effects are dominant and proto-sex chromosome frequencies resemble those in natural populations (Figure 4B and Supplementary Figure S14B). In contrast, $\rho_{MF}$ can be positive or negative for dominant fitness effects that maintain PSD regardless of the proto-sex chromosome frequency. However, as noted earlier, many of the cases where dominant fitness effects appear to maintain PSD are instead on a path to fixation that has not yet been reached at 1000 generations. When I only sample the dominant fitness effects that maintain PSD for 1,000,000 generations, I find that $\rho_{MF}<0$ (Supplementary Figure S15). Therefore, assuming 1,000,000 generations adequately approximates the equilibrium state, $\rho_{MF}<0$ when dominant fitness effects maintain PSD.

When fitness effects are overdominant or recessive, $\rho_{MF}$ does not provide evidence for sexually antagonistic selection. First, there is no clear pattern of $\rho_{MF}<0$ when fitness effects are recessive (Figure 4C and Supplementary Figure S14C). Second, overdominant fitness effects that produce the best-fitting genotype fitness arrays nearly all have $\rho_{MF}<0$ (Figure 4D and Supplementary Figure S14D), but this result is difficult to interpret because overdominant effects produce a concave relationship between the number of copies of a proto-Y chromosome and male fitness (*i.e.*, heterozygous males are defined as the most fit). Therefore, a simple rank order correlation (*i.e.*, $\rho_{MF}$) does not adequately capture the relationship between male and female fitness when the proto-Y chromosomes have overdominant effects in males.

### Negative intersexual fitness correlations are not caused by sexually antagonistic proto-Y chromosomes

**Figure 5:**
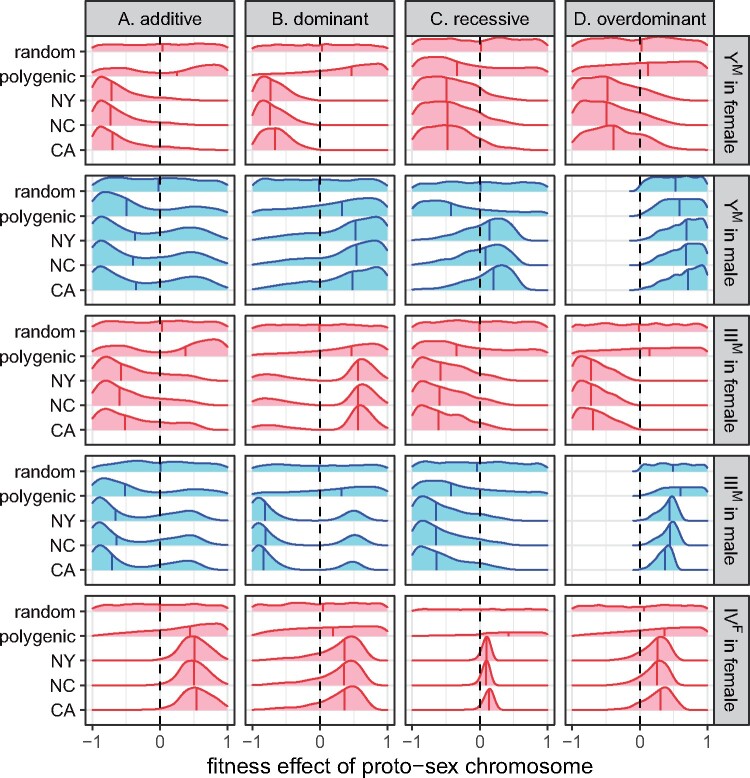
Smoothed histograms show the distributions of fitness effects of each proto-sex chromosome in each sex for 1000 random fitness arrays (random), fitness arrays that maintain PSD (polygenic), or the 1000 best-fitting fitness arrays for each population (CA, NC, or NY). Simulations were started with equal frequencies of all genotypes. The vertical line within each histogram shows the median, and dashed lines in each panel are at fitness value of 0. Fitness arrays were calculated assuming either (A) additive, (B) dominant, (C) recessive, or (D) overdominant fitness effects of the proto-Y chromosomes.

The negative $\rho_{MF}$ that I observe for additive and dominant fitness effects that maintain PSD (Figure 4 and Supplementary Figure S14) are suggestive of sexually antagonistic selection. A potential cause of $\rho_{MF}<0$ is sexually antagonistic effects of the proto-Y chromosomes if, for example, Y^M^ and III^M^ carry male-beneficial and female-detrimental alleles (Fisher 1931; Rice 1987). In contrast to this hypothesis, I do not observe a consistent signal of male-beneficial and female-detrimental proto-Y chromosomes when fitness effects are additive or dominant (Figure 5 and Supplementary Figure S16).

There is some evidence that Y^M^ is male-beneficial and female-detrimental when dominant fitness effects maintain PSD (Figure 5B and Supplementary Figure S16B). Additionally, both Y^M^ and III^M^ often confer a greater fitness benefit to males than females when dominant fitness effects maintain PSD (Supplementary Figures S17–S19). However, III^M^ does not tend to have male-beneficial and female-detrimental effects in the models with dominant fitness effects (Figure 5B and Supplementary Figure S16B). Therefore, when dominant fitness effects maintain PSD, the multi-chromosome genotypes tend to have sexually antagonistic fitness effects (Figure 4 and Supplementary Figures S14 and S15), but the individual chromosomes are not necessarily sexually antagonistic themselves.

In contrast to most expectations, additive fitness effects that maintain PSD often have female-beneficial and male-detrimental effects of the proto-Y chromosomes (Figure 5A and Supplementary Figure S16A). In addition, the proto-Y chromosomes do not consistently confer a greater fitness benefit to males than females when PSD is maintained by additive fitness effects (Supplementary Figures S17 and S18). Female-beneficial and male-detrimental Y chromosomes are opposite of the expected effects under most models of sex chromosome evolution (Rice 1987, 1996). However, some previous population genetic modeling has suggested the possibility of “feminization” of the Y chromosome (Cavoto *et al.* 2018).

Overdominant fitness effects that maintain proto-sex chromosomes at frequencies similar to those observed in natural populations have the strongest evidence of male-beneficial and female-detrimental proto-Y chromosomes (Figure 5D and Supplementary Figures S16D, S17D, and S18D). The overdominant model is constrained to require male-beneficial effects of the proto-Y chromosomes in heterozygotes, but female fitness effects can be beneficial or deleterious (see Supplementary Methods). Despite the full range of possible female fitness effects in the overdominant model, when proto-sex chromosomes are at frequencies similar to those in the CA, NC, or NY populations, the proto-Y chromosomes are almost always female-deterious (Figure 5D and Supplementary Figure S16D). These sexually antagonistic (female-deleterious and male-beneficial) effects of the Y^M^ and III^M^ proto-Y chromosomes in the overdominant model does not cause negative intersexual correlations of multi-chromosomal genotype fitness values (Figure 4 and Supplementary Figure S14) because heterozygous males are defined as the most fit.

### Female-beneficial effects of the proto-W chromosome

The IV^F^ proto-W chromosome is nearly always beneficial to females when PSD is maintained. This is the case regardless of the dominance of fitness effects of the proto-Y chromosomes, whether or not proto-sex chromosomes frequencies are similar to those in natural populations, and independently of the genotype frequencies used to start the simulations (Figure 5 and Supplementary Figure S16). There also tends to be a positive correlation between the fitness effect and frequency of IV^F^ when PSD is maintained (Supplementary Figures S20 and S21). A positive correlation indicates that the more female-beneficial the IV^F^ chromosome, the higher its frequency. The only exception to this rule is when dominant fitness effects maintain PSD for 1000 generations; however, as described earlier, these cases are on a path to fixation and therefore not at equilibrium.

## Discussion

### Dominance of fitness effects, sexually antagonistic sex chromosomes, and sexually antagonistic genotypes

I used multiple approaches to test if there is evidence of sexually antagonistic selection when PSD is maintained in my simulations. Sexual antagonism has previously been identified as a key component of the maintenance of PSD within populations (Bull 1983; Rice 1986; van Doorn and Kirkpatrick 2007; Meisel *et al.* 2016). A negative intersexual correlation of fitness across genotypes is a hallmark of sexually antagonistic selection (Chippindale *et al.* 2001; Rice and Chippindale 2001; Bonduriansky and Chenoweth 2009). I find strong evidence for $\rho_{MF}<0$ when dominant fitness effects maintain PSD (Figure 4B and Supplementary Figure S15). There is also some evidence that additive fitness effects that maintain PSD tend to have negative $\rho_{MF}$, although it is dependent on the frequency at which the proto-sex chromosomes are maintained (Figure 4A and Supplementary Figure S14A). However, neither dominant nor additive fitness effects that maintain PSD have male-beneficial and female-detrimental effects of all individual proto-Y chromosomes (Figure 5 and Supplementary Figure S16). Therefore, sexually antagonistic multi-chromosomal genotypes that maintain PSD can be emergent properties of cumulative fitness effects across chromosomes, without each chromosome having the same sexually antagonistic effects. This is evidence for a decoupling between single chromosome sexually antagonistic effects and sexual antagonism across multi-chromosome genotypes. It also suggests that testing for sexually antagonistic effects of individual proto-sex chromosomes may not be a sufficient assay of the role sexual antagonism plays in the maintenance of complex, multi-chromosomal PSD.

It has generally been observed that the dominance of fitness effects is an important parameter affecting the ability sexually antagonistic selection to maintain genetic variation (Connallon and Chenoweth 2019). For example, a sexually antagonistic allele is more likely to be maintained as a polymorphism if the sex-specific deleterious effects are recessive (Kidwell *et al.* 1977). In contrast, when fitness effects are dominant, stronger selection pressures are required to maintain the polymorphism (Kidwell *et al.* 1977). My work presented here contributes to the special case where the alleles under selection are on proto-sex chromosomes (as opposed to autosomes or heteromorphic sex chromosomes). Consistent with Kidwell *et al.* (1977), I find that dominant fitness effects that maintain PSD do indeed have strong selection in favor of or against proto-Y chromosomes in one sex (Figure 5 and Supplementary Figure S16).

In most of the models I consider here, the dominance of fitness effects of the proto-Y chromosomes is the same in both sexes. However, the dominance of fitness effects may be sex-specific (Barson *et al.* 2015; Grieshop and Arnqvist 2018). Such dominance reversals could be important for the maintenance of sexually antagonistic alleles (Spencer and Priest 2016; Connallon and Chenoweth 2019), including at sex-linked loci (Fry 2010). Therefore, it is possible that sex-specific dominance could create a broader range of fitness values than I observe to maintain PSD via sexually antagonistic selection. Consistent with this prediction, I observed PSD maintained most frequently when fitness effects of proto-Y chromosomes are overdominant in males and additive in females (see below for more discussion of overdominant fitness effects). Future work should further examine how sex-specific dominance affects the maintenance of PSD.

Sexually antagonistic proto-Y chromosomes require there to be segregating sexually antagonistic genetic variation. Male-beneficial and female-deleterious alleles are likely to accumulate on Y chromosomes because of their male-limited inheritance (Rice 1984). It is not clear, however, if that prediction applies when the Y chromosome can be transmitted through females in a complex PSD system. A more appropriate point of comparison for PSD systems may be autosomal and X-linked sexually antagonistic genetic variation, of which there is evidence for a substantial amount (Rice 1992; Chippindale *et al.* 2001; Calsbeek and Sinervo 2004; Fedorka and Mousseau 2004; Brommer *et al.* 2007; Foerster *et al.* 2007; Innocenti and Morrow 2010). The fact that such variation exists in a broad range of taxa suggests that sexually antagonistic proto-Y chromosomes may be possible in a PSD system.

My results are consistent with theory and data that suggest sexually antagonistic selection is important for the early evolution of sex chromosomes. For example, van Doorn and Kirkpatrick (2007, 2010) showed that selection on linked sexually antagonistic alleles can favor a new sex-determining locus because sex-limited inheritance can resolve the sexual conflict. Empirical studies have also identified sexually antagonistic variants on young Y and W chromosomes (Lindholm and Breden 2002; Roberts *et al.* 2009). In addition, once a new sex chromosome is established, fixation of additional alleles with sex-specific beneficial effects can be favored on the sex-specific Y or W chromosome (Abbott *et al.* 2017). It remains to be determined, however, whether young sex chromosomes are enriched for sexually antagonistic variants, if those variants are maintained by balancing selection, and if the sexually antagonistic alleles contribute to the maintenance of PSD. Investigating these problems could follow the approaches previously developed to test if balancing selection maintains sexually antagonistic alleles throughout the genome (*e.g.*, Dutoit *et al.* 2018; Ruzicka *et al.* 2019).

### Overdominance and the maintenance of PSD

Overdominant fitness effects of proto-Y chromosomes in males are more likely to maintain PSD than any other dominance scenario that I tested. Nearly half of the 4,000,000 overdominant fitness effects that I tested maintain all three proto-sex chromosomes at a frequency >0.1% for at least 1000 generations (Supplementary Figures S1A and S2A). In addition, nearly all of those overdominant fitness effects that maintain PSD for 1000 generations continue to do so for at least 1,000,000 generations (Supplementary Figures S4–S7), suggesting they are indeed stable polymorphisms at equilibrium. The frequencies with which overdominant fitness effects can maintain multiple polymorphic proto-sex chromosomes covers the full range of possible values, from close to 0 to nearly 1 (Figure 2D and Supplementary Figure S3D).

The extent of overdominant genetic variation—and balancing selection more generally—has been a topic of considerable debate in population genetics (Crow 1987; Delph and Kelly 2014; Fijarczyk and Babik 2015), with a few classic examples of heterozygote advantage (*e.g.*, Dobzhansky 1947; Allison 1956). Contemporary evidence for overdominant fitness effects in natural populations is mixed, including some work that suggests overdominance is rare (Andrés *et al.* 2009; Sellis *et al.* 2011, 2016; Hedrick 2012; Bitarello *et al.* 2018). Notably, in platyfish, which has a single chromosome PSD system, heterozygous males (XY) and females (WY or WX) have higher fitness than homozygotes (Orzack *et al.* 1980), providing evidence for overdominant fitness effects of sex chromosomes. Future work should evaluate the prevalence of proto-sex chromosomes with overdominant fitness effects in order to determine if the appropriate genetic variation exists for overdominance to maintain PSD more generally.

Regardless of the prevalence of overdominance in natural populations, it is one of the most straightforward mechanisms by which any polymorphism can be maintained (Hartl and Clark 2007). In my overdominant model, I assume that the proto-Y chromosomes carry male-beneficial additive or dominant alleles (Rice 1984, 1992), along with recessive deleterious alleles that have opposing effects equal in magnitude to the beneficial alleles (Charlesworth and Charlesworth 2000). Testing if these assumptions are biologically realistic would require measuring the magnitude and dominance of beneficial and deleterious alleles on proto-Y chromosomes. I also assume that the two homozygous genotypes (*i.e.*, XX and YY) have equal fitness (Table 2), which may not be biologically realistic. Future work should consider how different fitness values of the two homozygotes affects the ability of overdominance to maintain PSD.

When overdominant fitness effects in males maintain proto-sex chromosomes at frequencies most similar to those observed in natural populations, I find that they do so with female-deterious (additive) effects of the proto-Y chromosomes (Figure 5D and Supplementary Figure S16D). This is the strongest evidence I observe for sexually antagonistic fitness effects of individual proto-Y chromosomes (as opposed to multi-chromosomal genotypes) in any dominance scenario. It is also consistent with previous work demonstrating that overdominance in one sex can maintain genetic variants at individual loci even if there is directional selection against one allele in the other sex (Kidwell *et al.* 1977). In contrast to the additive and dominant fitness effects, there is no evidence for $\rho_{MF}<0$ when overdominant fitness effects maintain PSD (Figure 4 and Supplementary Figure S14). This provides additional evidence for a decoupling between single chromosome sexually antagonistic effects and sexual antagonism across multi-chromosome genotypes.

### Recombination on the proto-sex chromosomes and Y-linked alleles

My model makes assumptions about recombination on the sex chromosomes that may affect my conclusions about the maintenance of PSD. Specifically, I assume that there is no recombination between the male- or female-determining locus on each proto-Y or proto-W chromosome and the allele(s) under selection. Suppressed recombination is predicted to evolve in order to ensure that a sex-determining locus and sexually antagonistic alleles are inherited together on a young Y or W chromosome (Rice 1987). Suppressed recombination can also facilitate the invasion of a new sex determiner by increasing the effects of indirect selection on linked sexually antagonistic alleles, and it can further stabilize an ancestral sex chromosome through similar indirect effects (van Doorn and Kirkpatrick 2007, 2010). X-Y and Z-W recombination may be suppressed by chromosomal inversions that create tight genetic linkage between the sex-determining locus and sexually antagonistic alleles (Bergero and Charlesworth 2009; Wright et al. 2016).

Although there is no direct evidence for inversions or other recombination suppressors on the house fly sex chromosomes, male meiosis in flies is thought to occur without crossing over (Gethmann 1988). A lack of crossing over in males would prevent X-Y recombination in males without the need for sex-chromosome-specific modifiers. However, there is evidence for male recombination in house fly (Feldmeyer *et al.* 2010), which opens up the possibility for X-Y recombination. Furthermore, in the absence of Y-linked suppressors, X-Y recombination could also occur in the multiple female genotypes that carry a proto-Y chromosome (Table 1).

There are at least three consequences of X-Y or Z-W recombination that could affect how sex-specific selection maintains PSD in my model. First, my model assumes that all copies of each proto-Y and proto-W chromosome have identical fitness effects. This assumption is consistent with population genetics theory that predicts Y-linked variants cannot segregate as protected polymorphisms when there is no X-Y recombination (Clark 1987). In contrast to this prediction, however, there are numerous examples of Y-linked polymorphisms with phenotypic effects in *Drosophila*, where the X and Y do not recombine (Clark 1990; Zhang et al. 2000; Lemos *et al.* 2008, 2010; Griffin *et al.* 2015; Brown *et al.* 2020). Relevant to the house fly, these examples include Y-linked variation with temperature-dependent effects that are distributed across the species’ geographic range (Rohmer *et al.* 2004). Future work could incorporate context-dependent variation in fitness effects of each proto-Y and proto-W chromosome into my model to assess how segregating polymorphisms on the proto-sex chromosomes affect the maintenance of PSD.

A second consequence of X-Y recombination could affect how overdominant fitness effects maintain PSD in my model. I assume that overdominance arises from proto-Y chromosomes that carry recessive deleterious alleles. This assumption is based on the prediction that suppressed X-Y recombination leads to the “degeneration” of the Y chromosome via accumulation of (recessive) deleterious alleles because of Muller’s ratchet and hitchhiking (Charlesworth and Charlesworth 2000; Bachtrog 2013). In contrast, X-Y recombination in males and/or females could help purge recessive deleterious mutations from the Y^M^ and III^M^ chromosomes (Muller 1964; Felsenstein 1974). Moreover, there are multiple house fly genotypes that are homozygous for a proto-Y chromosome (Table 1), which would expose recessive deleterious mutations to selection. Selection in these proto-Y chromosome homozygotes would further remove recessive deleterious alleles from populations (Charlesworth *et al.* 1990; Barrett and Charlesworth 1991). Therefore, while overdominant fitness effects are likely to maintain PSD, proto-Y chromosomes may not possess the necessary alleles for such selection pressures to act upon. However, overdominance can also occur by other mechanisms besides degeneration of the Y chromosome and independent of accumulation of recessive deleterious Y-linked alleles (Sellis *et al.* 2011; Connallon and Clark 2014). These other causes of overdominance may allow for heterozygote fitness advantages to maintain PSD without degeneration of the proto-Y chromosomes.

Third, X-Y recombination would introduce X-linked alleles onto the proto-Y chromosomes. Male-limited inheritance of a Y chromosome is expected to lead to the accumulation of male-beneficial alleles in Y-linked genes, even if they have female-deleterious effects (Rice 1987, 1996). Conversely, female-biased inheritance of the X chromosome is expected to result in stronger selection on X-linked alleles in females if those alleles are partially dominant or segregating at moderate frequencies (Rice 1984; Charlesworth *et al.* 1987; Orr and Betancourt 2001), potentially feminizing the X chromosome. If there is X-Y recombination, those feminized X-linked alleles would be continuously introduced onto the Y chromosome, possibly feminizing the Y as well (Cavoto *et al.* 2018). This could provide a biological mechanism that allows for female-beneficial and male-detrimental effects of the proto-Y chromosomes that I predict when additive fitness effects maintain PSD (Figure 5A and Supplementary Figure S16A).

### Low frequency proto-sex chromosomes are unlikely to be maintained by selection

My simulations suggest that it is difficult for selection to maintain proto-sex chromosomes at low frequencies within populations. For example, the III^M^ proto-Y represents <3% of all third chromosomes in each of the three natural populations I considered (Hamm *et al.* 2005; Meisel *et al.* 2016). In my simulations, many of the selection pressures that maintain III^M^ at a frequency similar to those observed in each of the populations for 1000 generations eventually allow for the loss of III^M^ within 1,000,000 generations (Supplementary Figures S8 and S9). Additive fitness effects are especially likely to allow for the loss of III^M^. In addition, when PSD is maintained with proto-sex chromosomes at frequencies similar to natural populations, III^M^ tends to be at a higher frequency in my simulations than in any of the actual populations (Figure 2 and Supplementary Figure S3). III^M^ is also frequently lost when population size is finite, even when selection pressures exist that should maintain the polymorphism (Supplementary Figures S11 and S12). IV^F^ is also found at a low frequency (2–9% of all fourth chromosomes in the natural populations); it is similarly found at a higher frequency in the simulations than in the actual populations (Figure 2 and Supplementary Figure S3), and it is often lost in finite populations with selection (Supplementary Figures S11 and S12).

The behavior of III^M^ and IV^F^ in my simulations suggests that natural selection within populations may not be sufficient to maintain low frequency proto-sex chromosomes, and other factors may be required. Variation in proto-sex chromosome frequencies across house fly populations is suggestive of what those factors may be. In particular, spatially variable, temporally fluctuating, and other heterogeneous selection pressures can contribute to the maintenance of genetic variation across populations (Levene 1953; Hedrick 2006), including maintaining PSD (Bateman and Anholt 2017). The house fly Y^M^ and III^M^ proto-Y chromosomes are distributed along north-south clines on multiple continents (Denholm *et al.* 1986; Tomita and Wada 1989; Hamm *et al.* 2005; Kozielska *et al.* 2008), suggesting spatially heterogeneous selection pressures may be important for maintaining PSD across house fly populations. Migration between these populations could continuously introduce rare proto-Y and proto-W chromosomes, potentially overwhelming selection pressures that would otherwise allow for the loss of those proto-sex chromosomes within populations (Lenormand 2002; Tigano and Friesen 2016). Future work should directly model the effect of migration across populations with different selection pressures on the maintenance of PSD within populations.

### Proto-sex chromosomes are reminiscent of PARs, with some notable differences

Proto-sex chromosomes have some superficial similarities to PARs of heteromorphic sex chromosomes, within which there is X-Y (or Z-W) recombinatioin (Otto *et al.* 2011). Recombination in the PAR moves Y-linked alleles onto the X where they can be exposed to selection in females, reminiscent of how proto-Y chromosomes can be carried by females in a PSD system. Notably, PARs are capable of maintaining genetic variation, including sexually antagonistic alleles, under broader conditions than autosomes or even non-recombining regions of sex chromosomes (Jordan and Charlesworth 2012). These unique evolutionary dynamics of PARs are limited to loci close to the boundary with the non-recombining region of the sex chromosomes (Charlesworth *et al.* 2014). There is evidence for sexually antagonistic alleles in PARs from plants (Delph *et al.* 2010; Spigler *et al.* 2011) and fish (Tripathi *et al.* 2009; Kitano *et al.* 2009), but it is not yet clear whether PARs are enriched for sexually antagonistic alleles. Nonetheless, the possibility that PARs and proto-Y chromosomes are both hotspots of sexually antagonistic variation suggests they may experience similar selection pressures.

Despite the superficial similarities, there are important differences that make PARs imperfect analogs to proto-sex chromosomes. For example, when there is an epistatic dominant female-determining proto-W chromosome (as in house fly), the proto-Y chromosome itself can be transmitted through females. In contrast, the Y PAR cannot ever be carried by females. Instead, Y-linked PAR alleles must first recombine onto the X PAR in order to be carried by females. Moreover, males can be homozygous for the proto-Y chromosome, which is superficially similar to a male carrying an X PAR that has an ancestrally Y-linked allele. However, as above, this ancestral Y allele on the X PAR must recombine onto the X PAR, whereas, when there is PSD, homozygosity for the proto-Y occurs without X-Y recombination. Additional theoretical analyses are required to directly compare the invasion dynamics, fixation probabilities, and regions of stable polymorphisms across PARs and proto-sex chromosomes.

### Generalizing to even more complex PSD systems

I anticipate that many of the general patterns I observe here would be similar in other complex PSD systems with at least three different proto-sex chromosomes. For example, in a system with more than two proto-Y chromosomes, I expect to observe similar effects of dominance on the maintenance of PSD and sexual antagonism as in the system I modeled with two proto-Y chromosomes. In addition, if there are multiple proto-W chromosomes and a single (epistatic) proto-Y (*i.e.*, the opposite of the house fly system), I would expect similar patterns as I observe in the house fly system, but with the sexes reversed. These expectations could be tested in future work. It is not clear, however, what would be expected if there were both multiple proto-W and multiple proto-Y chromosomes in a population. It is worth noting that polygenic systems with many sex-determining loci are expected to be evolutionarily unstable (Rice 1986). Zebrafish may represent a promising model organism for combining empirical and theoretical approaches to make additional progress in understanding the maintenance of many sex-determining loci (Liew *et al.* 2012; Wilson et al. 2014).

## Conclusions

My results demonstrate that the maintenance of PSD depends on the dominance of fitness effects, which are further predictive of the sexually antagonistic effects of proto-sex chromosomes and genotypes. Notably, overdominant fitness effects are more likely to maintain PSD than fitness effects with other types of dominance. However, that conclusion requires at least one of two important assumptions: genetic variation with overdominant fitness effects must exist in natural populations, or proto-Y chromosomes must carry recessive deleterious alleles (in addition to additive or dominant beneficial alleles). My results also suggest that sexually antagonistic multi-chromosomal genotypes should be most prominent when dominant fitness effects (and to a lesser extent additive fitness effects) maintain PSD. This sexual antagonism is an emergent property of multi-chromosomal genotypes, and not intrinsic to the fitness effects of all individual proto-Y chromosomes. In contrast, when overdominant fitness effects in males maintain PSD, the proto-Y chromosomes tend to be male-beneficial and female-detrimental, but the multi-chromosomal genotypes do not have opposing fitness effects in males and females. Last, future work is needed to evaluate how X-Y (and Z-W) recombination, heterogeneous selection pressures, and migration across demes affect the ability of sex-specific selection pressures to maintain complex PSD, including in systems with other combinations of proto-sex chromosome.

## References

[jkab149-B1] Abbott JK , Nordén AK, Hansson B. 2017. Sex chromosome evolution: historical insights and future perspectives. Proc R Soc B. 284:20162806. 10.1098/rspb.2016.2806 PMC5443938 28469017

[jkab149-B2] Allison AC. 1956. The sickle-cell and haemoglobin C genes in some African populations. Ann Hum Genet. 21:67–89. 13340560

[jkab149-B3] Andrés AM , Hubisz MJ, Indap A, Torgerson DG, Degenhardt JD, et al 2009. Targets of balancing selection in the human genome. Mol Biol Evol. 26:2755–2764, 19713326 10.1093/molbev/msp190 PMC2782326

[jkab149-B4] Bachtrog D. 2013. Y-chromosome evolution: emerging insights into processes of Y-chromosome degeneration. Nat Rev Genet. 14:113–124. 23329112 10.1038/nrg3366 PMC4120474

[jkab149-B5] Bachtrog D , Mank JE, Peichel CL, Kirkpatrick M, Otto SP, et al; Tree of Sex Consortium 2014. Sex determination: why so many ways of doing it? PLoS Biol. 12:e1001899. 24983465 10.1371/journal.pbio.1001899 PMC4077654

[jkab149-B6] Barrett SC , Charlesworth D. 1991. Effects of a change in the level of inbreeding on the genetic load. Nature. 352:522–524. 1865906 10.1038/352522a0

[jkab149-B7] Barson NJ , Aykanat T, Hindar K, Baranski M, Bolstad GH, et al 2015. Sex-dependent dominance at a single locus maintains variation in age at maturity in salmon. Nature. 528:405–408. 26536110 10.1038/nature16062

[jkab149-B8] Bateman AW , Anholt BR. 2017. Maintenance of polygenic sex determination in a fluctuating environment: an individual-based model. J Evol Biol. 30:915–925. 28187242 10.1111/jeb.13054

[jkab149-B9] Beaumont MA , Zhang W, Balding DJ. 2002. Approximate Bayesian computation in population genetics. Genetics. 162:2025–2035. 12524368 10.1093/genetics/162.4.2025 PMC1462356

[jkab149-B10] Bergero R , Charlesworth D. 2009. The evolution of restricted recombination in sex chromosomes. Trends Ecol Evol. 24:94–102. 19100654 10.1016/j.tree.2008.09.010

[jkab149-B11] Beukeboom LW , Perrin N. 2014. The Evolution of Sex Determination. Oxford, UK: Oxford University Press.

[jkab149-B12] Bitarello BD , de Filippo C, Teixeira JC, Schmidt JM, Kleinert P, et al 2018. Signatures of long-term balancing selection in human genomes. Genome Biol Evol. 10:939–955. 29608730 10.1093/gbe/evy054 PMC5952967

[jkab149-B13] Bonduriansky R , Chenoweth SF. 2009. Intralocus sexual conflict. Trends Ecol Evol. 24:280–288. 19307043 10.1016/j.tree.2008.12.005

[jkab149-B14] Brommer JE , Kirkpatrick M, Qvarnström A, Gustafsson L. 2007. The intersexual genetic correlation for lifetime fitness in the wild and its implications for sexual selection. PLoS One. 2:e744. 17710144 10.1371/journal.pone.0000744 PMC1939732

[jkab149-B15] Brown EJ , Nguyen AH, Bachtrog D. 2020. The Y chromosome may contribute to sex-specific aging in *Drosophila*. Nat Ecol Evol. 4:853–862. 32313175 10.1038/s41559-020-1179-5 PMC7274899

[jkab149-B16] Bull JJ. 1983. Evolution of Sex Determining Mechanisms. Menlo Park, CA: Benjamin/Cummings.

[jkab149-B17] Calsbeek R , Sinervo B. 2004. Within-clutch variation in offspring sex determined by differences in sire body size: cryptic mate choice in the wild. J Evol Biol. 17:464–470. 15009280 10.1046/j.1420-9101.2003.00665.x

[jkab149-B18] Cavoto E , Neuenschwander S, Goudet J, Perrin N. 2018. Sex-antagonistic genes, XY recombination and feminized Y chromosomes. J Evol Biol. 31:416–427. 29284187 10.1111/jeb.13235

[jkab149-B19] Charlesworth B , Charlesworth D. 2000. The degeneration of Y chromosomes. Philos Trans R Soc Lond B Biol Sci. 355:1563–1572. 11127901 10.1098/rstb.2000.0717 PMC1692900

[jkab149-B20] Charlesworth B , Coyne JA, Barton NH. 1987. The relative rates of evolution of sex chromosomes and autosomes. Am Nat. 130:113–146.

[jkab149-B21] Charlesworth B , Jordan CY, Charlesworth D. 2014. The evolutionary dynamics of sexually antagonistic mutations in pseudoautosomal regions of sex chromosomes. Evolution. 68:1339–1350. 24476564 10.1111/evo.12364 PMC4289941

[jkab149-B22] Charlesworth D , Morgan MT, Charlesworth B. 1990. Inbreeding depression, genetic load, and the evolution of outcrossing rates in a multilocus system with no linkage. Evolution. 44:1469–1489. 28564321 10.1111/j.1558-5646.1990.tb03839.x

[jkab149-B23] Chippindale AK , Gibson JR, Rice WR. 2001. Negative genetic correlation for adult fitness between sexes reveals ontogenetic conflict in *Drosophila*. Proc Natl Acad Sci U S A. 98:1671–1675. 11172009 10.1073/pnas.041378098 PMC29315

[jkab149-B24] Clark AG. 1987. Variation in Y chromosome segregation in natural populations of *Drosophila melanogaster*. Genetics. 115:143–151. 3104134 10.1093/genetics/115.1.143 PMC1203050

[jkab149-B25] Clark AG. 1990. Two tests of Y chromosomal variation in male fertility of *Drosophila melanogaster*. Genetics. 125:527–534. 2116355 10.1093/genetics/125.3.527 PMC1204080

[jkab149-B26] Connallon T , Chenoweth SF. 2019. Dominance reversals and the maintenance of genetic variation for fitness. PLoS Biol. 17:e3000118. 30695026 10.1371/journal.pbio.3000118 PMC6368311

[jkab149-B27] Connallon T , Clark AG. 2014. Balancing selection in species with separate sexes: insights from Fisher’s geometric model. Genetics. 197:991–1006. 24812306 10.1534/genetics.114.165605 PMC4096376

[jkab149-B28] Connallon T , Singh ND, Clark AG. 2012. Impact of genetic architecture on the relative rates of X versus autosomal adaptive substitution. Mol Biol Evol. 29:1933–1942. 22319138 10.1093/molbev/mss057 PMC3408067

[jkab149-B29] Crow JF. 1987. Muller, Dobzhansky, and overdominance. J Hist Biol. 20:351–380.

[jkab149-B30] Delph LF , Kelly JK. 2014. On the importance of balancing selection in plants. New Phytol. 201:45–56. 23952298 10.1111/nph.12441 PMC3886833

[jkab149-B31] Delph LF , Arntz AM, Scotti-Saintagne C, Scotti I. 2010. The genomic architecture of sexual dimorphism in the dioecious plant *Silene latifolia*. Evolution. 64:2876–2886. 10.1111/j.1558-5646.2010.01048.x 20550575

[jkab149-B32] Denholm I , Franco MG, Rubini PG, Vecchi M. 1986. Geographical variation in house-fly (*Musca domestica* L.) sex determinants within the British Isles. Genet Res. 47:19–27.

[jkab149-B33] Dobzhansky T. 1947. Genetics of natural populations. XIV. A response of certain gene arrangements in the third chromosome of *Drosophila pseudoobscura* to natural selection. Genetics. 32:142–160. 20292738 10.1093/genetics/32.2.142 PMC1209369

[jkab149-B34] Dutoit L , Mugal CF, Bolívar P, Wang M, Nadachowska-Brzyska K, et al 2018. Sex-biased gene expression, sexual antagonism and levels of genetic diversity in the collared flycatcher (*Ficedula albicollis*) genome. Mol Ecol. 27:3572–3581. 30055065 10.1111/mec.14789

[jkab149-B35] Fedorka KM , Mousseau TA. 2004. Female mating bias results in conflicting sex-specific offspring fitness. Nature. 429:65–67. 15129280 10.1038/nature02492

[jkab149-B36] Feldmeyer B , Kozielska M, Kuijper B, Weissing FJ, Beukeboom LW, et al 2008. Climatic variation and the geographical distribution of sex-determining mechanisms in the housefly. Evol Ecol Res. 10:797–809.

[jkab149-B37] Feldmeyer B , Pen I, Beukeboom LW. 2010. A microsatellite marker linkage map of the housefly, *Musca domestica*: evidence for male recombination. Insect Mol. Biol. 19:575–581. 20491981 10.1111/j.1365-2583.2010.01016.x

[jkab149-B38] Felsenstein J. 1974. The evolutionary advantage of recombination. Genetics. 78:737–756. 4448362 10.1093/genetics/78.2.737 PMC1213231

[jkab149-B39] Fijarczyk A , Babik W. 2015. Detecting balancing selection in genomes: limits and prospects. Mol Ecol. 24:3529–3545. 25943689 10.1111/mec.13226

[jkab149-B40] Fisher RA. 1931. The evolution of dominance. Biol Rev Camb Philos Soc. 6:345–368.

[jkab149-B41] Foerster K , Coulson T, Sheldon BC, Pemberton JM, Clutton-Brock TH, et al 2007. Sexually antagonistic genetic variation for fitness in red deer. Nature. 447:1107–1110. 17597758 10.1038/nature05912

[jkab149-B42] Fry JD. 2010. The genomic location of sexually antagonistic variation: some cautionary comments. Evolution. 64:1510–1516. 19922443 10.1111/j.1558-5646.2009.00898.x PMC3654548

[jkab149-B43] Gethmann RC. 1988. Crossing over in males of higher Diptera (Brachycera). J Hered. 79:344–350. 31581763 10.1093/oxfordjournals.jhered.a110526

[jkab149-B44] Grieshop K , Arnqvist G. 2018. Sex-specific dominance reversal of genetic variation for fitness. PLoS Biol. 16:e2006810. 30533008 10.1371/journal.pbio.2006810 PMC6303075

[jkab149-B45] Griffin RM , Gall DL, Schielzeth H, Friberg U. 2015. Within-population Y-linked genetic variation for lifespan in *Drosophila melanogaster*. J Evol Biol. 28:1940–1947. 26230387 10.1111/jeb.12708

[jkab149-B46] Hamm RL. 2008. Exploring the population genetics of the house fly sex determining genes, M and F [PhD thesis]. Cornell University.

[jkab149-B47] Hamm RL , Scott JG. 2008. Changes in the frequency of Y^M^ versus III^M^ in the housefly, *Musca domestica* L., under field and laboratory conditions. Genet Res (Camb). 90:493–498. 19123967 10.1017/S0016672308009853

[jkab149-B48] Hamm RL , Scott JG. 2009. A high frequency of male determining factors in male *Musca domestica* (Diptera: Muscidae) from Ipswich, Australia. J Med Entomol. 46:169–172. 19198531 10.1603/033.046.0121

[jkab149-B49] Hamm RL , Meisel RP, Scott JG. 2015. The evolving puzzle of autosomal versus Y-linked male determination in *Musca domestica*. G3 (Bethesda) 5:371–384. 10.1534/g3.114.014795 PMC4349091 25552607

[jkab149-B50] Hamm RL , Shono T, Scott JG. 2005. A cline in frequency of autosomal males is not associated with insecticide resistance in house fly (Diptera: Muscidae). J Econ Entomol. 98:171–176. 15765679 10.1093/jee/98.1.171

[jkab149-B51] Hartl DL , Clark AG. 2007. Principles of Population Genetics. Sunderland, MA: Sinauer.

[jkab149-B52] Hediger M , Burghardt G, Siegenthaler C, Buser N, Hilfiker-Kleiner D, et al 2004. Sex determination in *Drosophila melanogaster* and *Musca domestica* converges at the level of the terminal regulator *doublesex*. Dev Genes Evol. 214:29–42. 14673649 10.1007/s00427-003-0372-2

[jkab149-B53] Hediger M , Henggeler C, Meier N, Perez R, Saccone G, et al 2010. Molecular characterization of the key switch *F* provides a basis for understanding the rapid divergence of the sex-determining pathway in the housefly. Genetics. 184:155–170. 19841093 10.1534/genetics.109.109249 PMC2815913

[jkab149-B54] Hedrick PW. 2006. Genetic polymorphism in heterogeneous environments: the age of genomics. Annu Rev Ecol Evol Syst. 37:67–93.

[jkab149-B55] Hedrick PW. 2012. What is the evidence for heterozygote advantage selection? Trends Ecol Evol. 27:698–704. 22975220 10.1016/j.tree.2012.08.012

[jkab149-B56] Innocenti P , Morrow EH. 2010. The sexually antagonistic genes of *Drosophila melanogaster*. PLoS Biol. 8:e1000335. 20305719 10.1371/journal.pbio.1000335 PMC2838750

[jkab149-B57] Jordan CY , Charlesworth D. 2012. The potential for sexually antagonistic polymorphism in different genome regions. Evolution. 66:505–516. 22276544 10.1111/j.1558-5646.2011.01448.x

[jkab149-B58] Kallman KD. 1973. The sex-determining mechanism of the platyfish, *Xiphophorus maculatus.* In: Genetics and Mutagenesis of Fish. Berlin Heidelberg: Springer. p. 19–28. pp.

[jkab149-B59] Kidwell JF , Clegg MT, Stewart FM, Prout T. 1977. Regions of stable equilibria for models of differential selection in the two sexes under random mating. Genetics. 85:171–183. 838269 10.1093/genetics/85.1.171 PMC1213615

[jkab149-B60] Kitano J , Ross JA, Mori S, Kume M, Jones FC, et al 2009. A role for a neo-sex chromosome in stickleback speciation. Nature. 461:1079–1083. 19783981 10.1038/nature08441 PMC2776091

[jkab149-B61] Kozielska M , Feldmeyer B, Pen I, Weissing FJ, Beukeboom LW. 2008. Are autosomal sex-determining factors of the housefly (*Musca domestica*) spreading north? Genet Res (Camb). 90:157–165. 18426619 10.1017/S001667230700907X

[jkab149-B62] Kozielska M , Pen I, Beukeboom LW, Weissing FJ. 2006. Sex ratio selection and multi-factorial sex determination in the housefly: a dynamic model. J Evol Biol. 19:879–888. 16674584 10.1111/j.1420-9101.2005.01040.x

[jkab149-B63] Lemos B , Araripe LO, Hartl DL. 2008. Polymorphic Y chromosomes harbor cryptic variation with manifold functional consequences. Science. 319:91–93. 18174442 10.1126/science.1148861

[jkab149-B64] Lemos B , Branco AT, Hartl DL. 2010. Epigenetic effects of polymorphic Y chromosomes modulate chromatin components, immune response, and sexual conflict. Proc Natl Acad Sci U S A. 107:15826–15831. 20798037 10.1073/pnas.1010383107 PMC2936610

[jkab149-B65] Lenormand T. 2002. Gene flow and the limits to natural selection. Trends Ecol Evol. 17:183–189.

[jkab149-B66] Levene H. 1953. Genetic equilibrium when more than one ecological niche is available. Am Nat. 87:331–333.

[jkab149-B67] Liew WC , Bartfai R, Lim Z, Sreenivasan R, Siegfried KR, et al 2012. Polygenic sex determination system in zebrafish. PLoS One. 7:e34397. 22506019 10.1371/journal.pone.0034397 PMC3323597

[jkab149-B68] Lindholm A , Breden F. 2002. Sex chromosomes and sexual selection in poeciliid fishes. Am Nat. 160:S214–24. 18707478 10.1086/342898

[jkab149-B69] McDonald I. C , Evenson P, Nickel CA, Johnson OA. 1978. House fly genetics: isolation of a female determining factor on chromosome 4. Ann Entomol Soc Am. 71:692–694.

[jkab149-B70] Meier N , Käppeli SC, Hediger Niessen M, Billeter J-C, Goodwin SF, et al 2013. Genetic control of courtship behavior in the housefly: evidence for a conserved bifurcation of the sex-determining pathway. PLoS One. 8:e62476. 23630634 10.1371/journal.pone.0062476 PMC3632534

[jkab149-B71] Meisel RP , Davey T, Son JH, Gerry AC, Shono T, et al 2016. Is multifactorial sex determination in the house fly, *Musca domestica* (L.), stable over time? J Hered. 107:615–625. 27540102 10.1093/jhered/esw051

[jkab149-B72] Meisel RP , Gonzales CA, Luu H. 2017. The house fly Y Chromosome is young and minimally differentiated from its ancient X Chromosome partner. Genome Res. 27:1417–1426. 28619849 10.1101/gr.215509.116 PMC5538557

[jkab149-B73] Moore EC , Roberts RB. 2013. Polygenic sex determination. Curr Biol. 23:R510–R512. 23787041 10.1016/j.cub.2013.04.004

[jkab149-B74] Muller HJ. 1964. The relation of recombination to mutational advance. Mutat Res. 1:2–9. 10.1016/0027-5107(64)90047-8 14195748

[jkab149-B75] Orr HA. 2010. The population genetics of beneficial mutations. Philos Trans R Soc B. 365:1195–1201. 10.1098/rstb.2009.0282 PMC2871816 20308094

[jkab149-B76] Orr HA , Betancourt AJ. 2001. Haldane’s sieve and adaptation from the standing genetic variation. Genetics. 157:875–884. 11157004 10.1093/genetics/157.2.875 PMC1461537

[jkab149-B77] Orzack SH , Sohn JJ, Kallman KD, Levin SA, Johnston R. 1980. Maintenance of the three sex chromosome polymorphism in the platyfish *Xiphophorus maculatus*. Evolution. 34:663–672. 28563992 10.1111/j.1558-5646.1980.tb04005.x

[jkab149-B78] Otto SP , Pannell JR, Peichel CL, Ashman T-L, Charlesworth D, et al 2011. About PAR: the distinct evolutionary dynamics of the pseudoautosomal region. Trends Genet. 27:358–367. 21962971 10.1016/j.tig.2011.05.001

[jkab149-B79] Rice WR. 1984. Sex chromosomes and the evolution of sexual dimorphism. Evolution. 38:735–742. 28555827 10.1111/j.1558-5646.1984.tb00346.x

[jkab149-B80] Rice WR. 1986. On the instability of polygenic sex determination: the effect of sex-specific selection. Evolution. 40:633–639. 28556317 10.1111/j.1558-5646.1986.tb00514.x

[jkab149-B81] Rice WR. 1987. The accumulation of sexually antagonistic genes as a selective agent promoting the evolution of reduced recombination between primitive sex chromosomes. Evolution. 41:911–914. 28564364 10.1111/j.1558-5646.1987.tb05864.x

[jkab149-B82] Rice WR. 1992. Sexually antagonistic genes: experimental evidence. Science. 256:1436–1439. 1604317 10.1126/science.1604317

[jkab149-B83] Rice WR. 1996. Evolution of the Y sex chromosome in animals. Bioscience. 46:331–343.

[jkab149-B84] Rice WR , Chippindale AK. 2001. Intersexual ontogenetic conflict. J Evol Biol. 14:685–693.

[jkab149-B85] Roberts RB , Ser JR, Kocher TD. 2009. Sexual conflict resolved by invasion of a novel sex determiner in Lake Malawi cichlid fishes. Science. 326:998–1001. 19797625 10.1126/science.1174705 PMC3174268

[jkab149-B86] Rohmer C , David JR, Moreteau B, Joly D. 2004. Heat induced male sterility in *Drosophila melanogaster*: adaptive genetic variations among geographic populations and role of the Y chromosome. J Exp Biol. 207:2735–2743. 15235002 10.1242/jeb.01087

[jkab149-B87] Ruzicka F , Hill MS, Pennell TM, Flis I, Ingleby FC, et al 2019. Genome-wide sexually antagonistic variants reveal long-standing constraints on sexual dimorphism in fruit flies. PLoS Biol. 17:e3000244. 31022179 10.1371/journal.pbio.3000244 PMC6504117

[jkab149-B88] Scott JG , Leichter CA, Rinkevich FD, Harris SA, Su C, et al 2013. Insecticide resistance in house flies from the United States: resistance levels and frequency of pyrethroid resistance alleles. Pestic Biochem Physiol. 107:377–384. 24267700 10.1016/j.pestbp.2013.10.006

[jkab149-B89] Scott JG , Warren WC, Beukeboom LW, Bopp D, Clark AG, et al 2014. Genome of the house fly, *Musca domestica* L., a global vector of diseases with adaptations to a septic environment. Genome Biol. 15:466. 25315136 10.1186/s13059-014-0466-3 PMC4195910

[jkab149-B90] Sellis D , Callahan BJ, Petrov DA, Messer PW. 2011. Heterozygote advantage as a natural consequence of adaptation in diploids. Proc Natl Acad Sci U S A. 108:20666–20671. 22143780 10.1073/pnas.1114573108 PMC3251125

[jkab149-B91] Sellis D , Kvitek DJ, Dunn B, Sherlock G, Petrov DA. 2016. Heterozygote advantage is a common outcome of adaptation in *Saccharomyces cerevisiae*. Genetics. 203:1401–1413. 27194750 10.1534/genetics.115.185165 PMC4937471

[jkab149-B92] Sharma A , Heinze SD, Wu Y, Kohlbrenner T, Morilla I, et al 2017. Male sex in houseflies is determined by *Mdmd*, a paralog of the generic splice factor gene *CWC22*. Science. 356:642–645. 28495751 10.1126/science.aam5498

[jkab149-B93] Son JH , Meisel RP. 2021. Gene-level, but not chromosome-wide, divergence between a very young house fly proto-Y chromosome and its homologous proto-X chromosome. Mol Biol Evol. 38:606–618. 32986844 10.1093/molbev/msaa250 PMC7826193

[jkab149-B94] Spencer HG , Priest NK. 2016. The evolution of sex-specific dominance in response to sexually antagonistic selection. Am Nat. 187:658–666. 27104997 10.1086/685827

[jkab149-B95] Spigler RB , Lewers KS, Ashman T-L. 2011. Genetic architecture of sexual dimorphism in a subdioecious plant with a proto-sex chromosome. Evolution. 65:1114–1126. 21062281 10.1111/j.1558-5646.2010.01189.x

[jkab149-B96] Tigano A , Friesen VL. 2016. Genomics of local adaptation with gene flow. Mol Ecol. 25:2144–2164. 26946320 10.1111/mec.13606

[jkab149-B97] Tomita T , Wada Y. 1989. Multifactorial sex determination in natural populations of the housefly (*Musca domestica*) in Japan. Jpn J Genet. 64:373–382.

[jkab149-B98] Tripathi N , Hoffmann M, Willing E-M, Lanz C, Weigel D, et al 2009. Genetic linkage map of the guppy, Poecilia reticulata, and quantitative trait loci analysis of male size and colour variation. Proc Biol Sci. 276:2195–2208. 19324769 10.1098/rspb.2008.1930 PMC2677598

[jkab149-B99] van Doorn GS , Kirkpatrick M. 2007. Turnover of sex chromosomes induced by sexual conflict. Nature. 449:909–912. 17943130 10.1038/nature06178

[jkab149-B100] van Doorn GS , Kirkpatrick M. 2010. Transitions between male and female heterogamety caused by sex-antagonistic selection. Genetics. 186:629–645. 20628036 10.1534/genetics.110.118596 PMC2954476

[jkab149-B101] Wilkins AS. 1995. Moving up the hierarchy: a hypothesis on the evolution of a genetic sex determination pathway. Bioessays. 17:71–77. 7702596 10.1002/bies.950170113

[jkab149-B102] Wilson CA , High SK, McCluskey BM, Amores A, Yan Y-L, et al 2014. Wild sex in zebrafish: loss of the natural sex determinant in domesticated strains. Genetics. 198:1291–1308. 25233988 10.1534/genetics.114.169284 PMC4224167

[jkab149-B103] Wright AE , Dean R, Zimmer F, Mank JE. 2016. How to make a sex chromosome. Nat. Commun. 7:12087. 27373494 10.1038/ncomms12087 PMC4932193

[jkab149-B104] Zhang P , Timakov B, Stankiewicz RL, Turgut IY. 2000. A trans-activator on the *Drosophila* Y chromosome regulates gene expression in the male germ line. Genetica. 109:141–150. 11293789 10.1023/a:1026504721067

